# *SMS2*, a Novel Allele of *OsINV3*, Regulates Grain Size in Rice

**DOI:** 10.3390/plants13091219

**Published:** 2024-04-28

**Authors:** Jianzhi Huang, Zelong Zhou, Ying Wang, Jing Yang, Xinyue Wang, Yijun Tang, Ran Xu, Yunhai Li, Lian Wu

**Affiliations:** 1 School of Breeding and Multiplication (Sanya Institute of Breeding and Multiplication), Hainan University, Sanya 572025, Chinaxuran@hainanu.edu.cn (R.X.); 2 Department of Resources and Environment, Zunyi Normal College, Ping An Avenue, Xinpu New District, Zunyi 563006, China; 3 State Key Laboratory of Plant Cell and Chromosome Engineering, CAS Centre for Excellence in Molecular Plant Biology, Institute of Genetics and Developmental Biology, Chinese Academy of Sciences, Beijing 100101, China; yhli@genetics.ac.cn

**Keywords:** grain size, yield, rice, *SMS2*, *OsINV3*

## Abstract

Grain size has an important effect on rice yield. Although several key genes that regulate seed size have been reported in rice, their molecular mechanisms remain unclear. In this study, a rice small grain size 2 (*sms2*) mutant was identified, and MutMap resequencing analysis results showed that a 2 bp insertion in the second exon of the *LOC_Os02g01590* gene resulted in a grain length and width lower than those of the wild-type Teqing (TQ). We found that *SMS2* encoded vacuolar acid invertase, a novel allele of *OsINV3*, which regulates grain size. GO and KEGG enrichment analyses showed that *SMS2* was involved in endoplasmic reticulum protein synthesis, cysteine and methionine metabolism, and propionic acid metabolism, thereby regulating grain size. An analysis of sugar content in young panicles showed that *SMS2* reduced sucrose, fructose, and starch contents, thus regulating grain size. A haplotype analysis showed that Hap2 of *SMS2* had a longer grain and was widely present in *indica* rice varieties. Our results provide a new theoretical basis for the molecular and physiological mechanisms by which *SMS2* regulates grain size.

## 1. Introduction

Plant organ size is a crucial aspect of plant environmental adaptability, and it is determined by both the internal genotype and the external environment. The size and shape of organs vary greatly among species, while the size of organs is relatively consistent among individuals of the same species. This indicates that plant organ size and shape are strictly regulated by genetics [1]. In addition, plant organ size is often affected by changes in external environmental conditions. Plants must feel and adapt to changes in the external environment as well as maintain internal metabolism balance and stability. Complex signaling networks may regulate the size of organs to some extent.

Rice grain size regulation is a complex biological process that is affected by internal and external factors. Internal factors include genes and various regulatory factors, while external factors include environmental factors such as light, temperature, moisture, and nutrition. In general, various external factors and regulatory factors mostly affect plant endogenous hormone levels, gene expression or protein activity, and localization, which are indirectly involved in regulating grain size [2]. Rice grain shape is closely related to yield and quality traits, and it has become an important target trait in the high-yield and high-quality molecular breeding of rice [3]. The molecular network of grain size regulation has begun to take shape, but the specific biological mechanism remains unclear. Further elucidating the mechanism of grain size regulation can provide an important theoretical basis and technical support for the improvement of rice germplasm and production efficiency. Therefore, an identification and functional analysis of grain shape genes is one of the most important areas of research in rice genetics.

In recent years, an increasing number of grain shape quantitative trait loci (QTLs)/genes have been cloned in rice [4], such as *GW2* [5], *GS2* [6], *TGW2* [7], *OsLG3* [8], *LGY3* [9], *GS3* [10], *GL3.1* [11], *GS5* [12], *GW5* [13], *GL5* [14], *GW6* [15], *TGW6* [16], *GW8* [17], and *GS9* [18]. These cloned genes mainly involve plant hormones, the ubiquitin–proteasome pathway, the MAPK signaling pathway, the G-protein signaling pathway, and transcription factors, thereby regulating rice grain size [3,19]. For instance, *qGL3/OsPPKL1* negatively regulates the grain length and weight of rice, and it regulates the proliferation and elongation of spike cells through hormone signaling [11]. *GW2* encodes E3 ubiquitin ligase, which increases rice grain width and weight by altering cell proliferation [5]. *GS2/GL2* negatively regulates rice grain size and weight through hormone signaling and transcription factor pathways [6]. OsMKKK10 can activate OsMKK4 and OsMAPK6 in turn, forming a cascade reaction to positively regulate rice grain size and weight [20]. Three atypical G-protein γ subunits, namely, GS3, DEP1, and GGC2, coexist in rice, and their regulation of grain size is different due to the variation in the protein C-terminal domain [21]. *GS9* negatively regulates grain length and width in rice via transcription factor signaling [18]. Recent studies have found that the E3 ubiquitin ligase CLG1 can ubiquitinate the G-protein gamma subunit GS3, act upstream of GS3 to mediate its degradation, and regulate rice grain length by balancing G-protein signaling [22]. The transcription factor SPL12 and GW5 co-evolve to regulate grain width in rice [23]. Recently, it was found that *qGL3* can phosphorylate OsVIL1 and participate in the BR pathway to change grain traits [24]. In addition, mutations in the PPKL1-specific amino acid D364 were found to activate the cytokinin response, thereby increasing grain size [25]. SMG4 interacts with COPII vesicle components and members of the cytochrome P450 subfamily CYP78A to regulate rice grain size [26]. FGW1 may interact with GF14f to influence grain filling and, thus, regulate grain size [27]. *GSW3* regulates grain length and width by promoting cell division and growth [28]. The *TGW3* pathway mediates auxin signaling to regulate rice grain yield [29]. There are different genetic interaction relationships among grain shape genes. The effect of the interaction between grain shape genes on yield and rice quality can be realized through the rational collocation of an excellent genotype combination. A previous study found that the aggregation of the long-grain allele *gs9* and the excellent upright panicle allele *dep1* could improve the appearance quality of rice without changing the plant type or panicle shape of the upright panicle quality [30]. The polymerization of the long-grain allele *gs10* with *GW5* could improve the appearance quality of rice [31]. The polymerization of *gs3*, *gl3.1*, *gw5*, and *GW7* could significantly improve the appearance quality of rice [32]. Additionally, the *gs3*, *GS5*, *gw5*, and *GL7* alleles could be aggregated to form Guangdong high-quality varieties with an excellent appearance quality [33]. Therefore, the continuous research on the interaction between grain shape genes and the exploration of gene combinations suitable for breeding promotion can promote the rapid cultivation of high-yield and high-quality rice.

MutMap is a forward genetics gene mapping strategy and a genetic analysis method developed based on whole-genome sequencing (WGS), and it is a powerful tool for the rapid discovery and utilization of excellent genetic variants [34]. With the development of high-throughput second-generation sequencing technology and the rapid reduction in sequencing cost, the genome sequences of an increasing number of species have been analyzed; thus, the MutMap-based method has a large application space. To date, the MutMap method has been used to locate genes responsible for important agronomic traits in rice, such as plant height [35,36], grain size [37], and salt tolerance [38], which indicates that the MutMap method has become an important tool for forward genetics research.

Sucrose is the main carbohydrate transport form. It is produced by photosynthesis in the leaf cytoplasm and is transported to storage tissues through the phloem to provide energy for plant growth and development. In addition, studies have found that sucrose can also act as a signaling molecule and interact with plant hormones to affect plant growth, tissue differentiation, organ development, flowering, and fruiting [39,40]. The decomposition of sucrose mainly relies on two enzymes: sucrose synthase (SUS) and invertase (INV). According to its subcellular localization and optimal pH value, INV can be divided into acidic cell wall invertase (CWIN), acidic vacuolar invertase (VIN), and neutral/basic cytoplasmic invertase (CIN) [41]. VIN plays an important role in sugar accumulation and cell elongation. On the one hand, it provides an energy source for cell growth, and, on the other hand, it promotes cell elongation by regulating osmotic pressure. Therefore, the rapidly expanding parts of plants have high VIN activity [42]. Nine CWINs, two VINs, and eight CINs have been identified in rice [43]. CWINs regulate the transport and distribution of assimilate in rice, thereby regulating rice yield. For example, *OsCIN1* was discovered to regulate grain filling [44]; *OsCIN2* was found to control grain size [45]. In rice, *OsINV2* and *OsINV3* affect grain filling, grain size, and yield by altering rice sugar metabolism, including sugar composition, transport, and starch accumulation [46,47,48,49]. Although the role of VINs in rice has been reported in many articles, the molecular mechanisms underlying the regulation of rice grain size by VINs are largely unknown.

In this study, we aimed to locate the candidate gene *SMS2* of small-grain mutants using MutMap technology, then analyze the molecular mechanism by which *SMS2* regulates grain size in rice using RNA-seq technology, and finally determine the dominant haplotype of *SMS2* through a haplotype analysis. The above analyses provide more information for the analysis of the molecular mechanism of rice grain size formation.

## 2. Results

### 2.1. Characterization of sms2 Mutant

**Figure 1:**
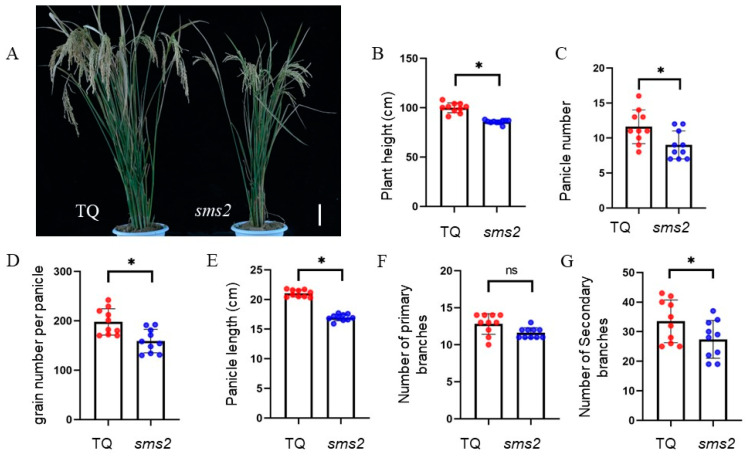
Comparison of yield traits between TQ and *sms2*. (**A**) Plants of TQ and *sms2*; bar = 10 cm. (**B**) Plant height of TQ and *sms2*. (**C**) Panicle number of TQ and *sms2*. (**D**) Grain number per panicle of TQ and *sms2*. (**E**) Panicle length of TQ and *sms2*. (**F**) Number of primary branches of TQ and *sms2*. (**G**) Number of secondary branches of TQ and *sms2*. The data are the mean ± S.D (*n* = 10 plants). * *p* < 0.05; significant differences were based on 2-tailed *t*-test. ns indicates no significant difference. The red and blue dots indicate the individual values of TQ and *sms2*, respectively.

In the background of the indica rice variety Teqing (TQ), a series of mutant materials with grain size changes were selected by means of EMS. One of the mutations, named *sms2*, was characterized by a reduced grain size. The yield characteristics of TQ and *sms2* were measured at the maturity stage. Compared with TQ, the plant height (Figure 1A,B), panicle number (Figure 1C), grain number per panicle (Figure 1D), panicle length (Figure 1E), and number of secondary branches (Figure 1G) of *sms2* were significantly decreased, but the number of primary branches showed no significant difference (Figure 1F). The F_1_ plants of the TQ and *sms2* hybrid had heterosis, and their height was higher than that of TQ and *sms2*.

**Figure 2:**
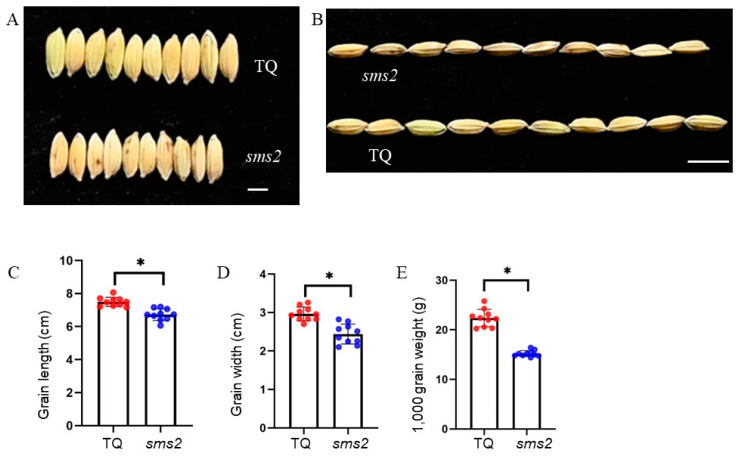
Comparison of grain size traits between TQ and *sms2*. (**A**,**B**) Grain size of TQ and *sms2*; bar = 3 mm (2A), bar = 8 mm (2B). (**C**) Grain length of TQ and *sms2*. (**D**) Grain width of TQ and *sms2*. (**E**) The 1000-grain weight of TQ and sms2. The data are the mean ± S.D (*n* = 10 plants). * *p* < 0.05; significant differences were based on 2-tailed *t*-test. The red and blue dots indicate the individual values of TQ and *sms2*, respectively.

Compared with TQ, the grain length, grain width, and 1000-grain weight of the *sms2* mutant were significantly reduced (Figure 2A–E). The measurement results showed that the average seed length of TQ was about 7.49 mm and that of the *sms2* mutant was 6.72 mm, showing a decrease of 10.2% compared with that of the wild type. Compared with TQ (2.96 mm), the grain width of *sms2* (2.43 mm) was significantly decreased by 18.2%, resulting in a significant decrease in 1000-grain weight (32.09%). The grain length of the F_1_ plants hybridized with TQ and SMS2 was larger than that of *sms2* but smaller than that of TQ. There was no significant difference in grain width among the three.

### 2.2. Identification of SMS2 Gene

**Figure 3:**
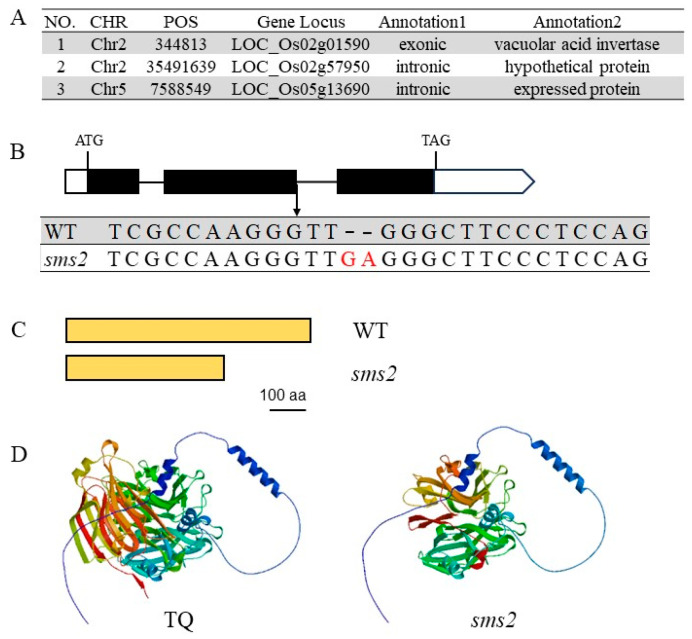
Identification of the *sms2* mutation. (**A**) Cloning of *SMS2* gene using MutMap. (**B**) The *SMS2* gene structure, showing *SMS2* mutation site. (**C**) The SMS2 protein structure; *sms2* mutates to form a truncated mutant protein. (**D**) Three-dimensional structure of SMS2 protein. In the figure, the red font GA is the base position inserted by the mutant.

The Mutmap method was used to identify the *sms2* mutation. The *sms2* mutant was crossed with TQ to generate an F_2_ population, and the segregation ratio of the F_2_ population showed that *sms2* is a single recessive mutation. Thirty F_2_ plants with a small-grain phenotype were pooled for whole-genome resequencing, and the genome of TQ was resequenced as a control. We identified three candidate SNPs’ (single-nucleotide polymorphisms) sites closely associated with phenotypes (Figure 3A). One SNP was located in the gene’s coding region. Gene annotation showed that the SNPs were located at the end of the second exon of *LOC_Os02g01590*, a gene encoding vacuolar acid invertase. Sequence alignment revealed that the *sms2* mutant was caused by the insertion of two G-A bases in the second exon of the gene (Figure 3B), resulting in the formation of a truncated protein (from 661 amino terminus to 428 amino acids) (Figure 3C). The results of protein 3D structure prediction showed that there were significant differences between TQ and *sms2* (Figure 3D). After an analysis of gene function, *LOC_Os02g01590* was discovered to be a cloned *OsINV3* gene. This gene controls cell expansion to regulate rice grain size. These results indicate that *LOC_Os02g01590* is a candidate gene for *SMS2*.

### 2.3. GO and KEGG Analyses of Grain Size Regulation via SMS2

**Figure 4:**
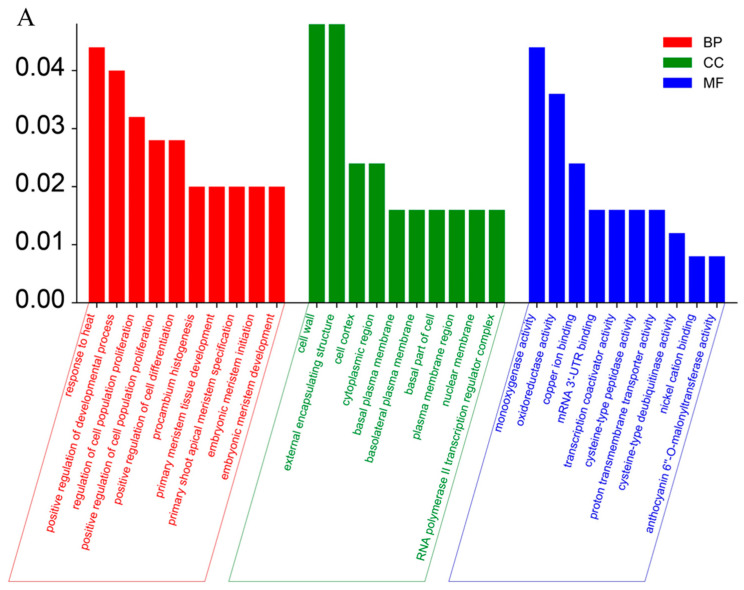

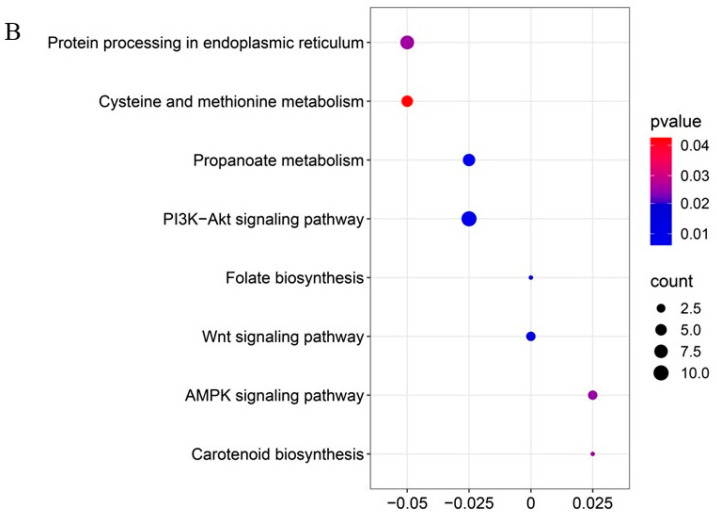
RNA-Seq analysis of regulation of grain size by *SMS2* gene. (**A**) Gene Ontology analysis. (**B**) KEGG analysis.

In order to investigate the potential molecular basis by which *SMS2* regulates grain size, transcriptomics was used to analyze the gene expression of TQ and *sms2* young panicles. GO and KEGG enrichment analyses were performed according to differential functional genes. The results of the GO analysis showed that there were 30 GO terms significantly enriched with differentially expressed genes. There were 10 significantly enriched GO terms in the biological process category. There were 10 significantly enriched GO terms in the cellular component category. There were 10 significantly enriched GO terms in the molecular function category. The top three GO terms of biological process enrichment were heat stress response, growth and development regulation, and cell proliferation regulation. The top three GO terms enriched in the cellular component category were cell wall, external encapsulating structure, and cell cortex. The top three GO terms of molecular function enrichment were monooxygenase activity, oxidoreductase activity, and copper ion binding (Figure 4A). The KEGG enrichment analysis showed that a total of eight metabolic processes were significantly enriched. The top four metabolic pathways significantly enriched were endoplasmic reticulum protein synthesis, cysteine and methionine metabolism, propanoate metabolism, and the PI3K-Akt signaling pathway, indicating that the metabolic pathway by which *SMS2* regulates grain size is most likely to play a role through the above four significantly enriched metabolic pathways. As the number of differentially expressed genes enriched in the other four metabolic pathways was small, the possibility of their participating in the regulation of grain size was low (Figure 4B). These findings suggest that *SMS2* may mediate these processes to regulate grain size.

We focused on RNA-seq data to analyze the expression of the key genes controlling grain size, hormones, the cell cycle, and cell division in TQ and the *sms2* mutant. The analysis results showed that there were significant differences in the expression level of the *GL7* gene in TQ and *sms2*; we deemed the reason for the higher expression level of the *GL7* gene in the *sms2* mutant to be worth exploring. The 10 genes identified were *LOC_Os03g02470*, *LOC_Os09g27820*, *LOC_Os03g43100*, *LOC_Os06g15430*, *LOC_Os09g27744*, *LOC_Os06g29360*, *LOC_Os06g13690*, *LOC_Os07g02500*, *LOC_Os10g33030*, and *LOC_Os07g03015*. It was found that *LOC_Os09g27820* encoded the aminocyclopropane-1-carboxylate oxidase gene, and its high expression was associated with internode elongation in deep-water rice under flooding conditions [50], but its role in development was not clear. Therefore, we speculate that *LOC_Os09g27820* may be an important regulatory factor in grain development and can be used as the focus of further research. The other nine genes, all of which encode unknown proteins, also need to be identified for their role in grain size. The qRT-PCR results showed that the gene expression trend of the four genes was basically consistent with the transcriptome sequencing results, thus demonstrating the reliability of the transcriptome sequencing results.

### 2.4. SMS2 Reduces Sugar and Starch Accumulation and Invertase Activity

**Figure 5:**
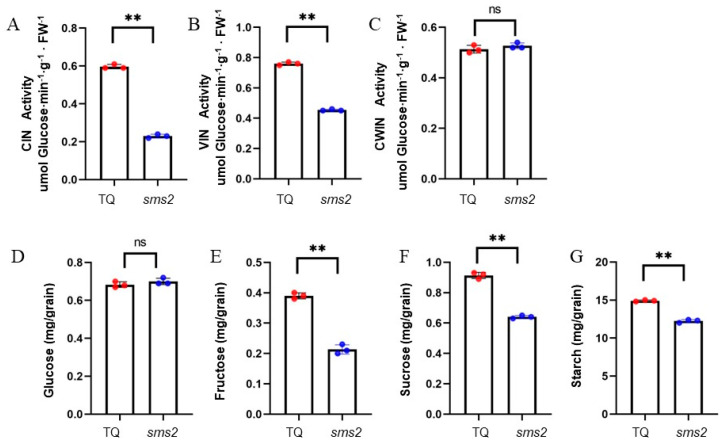
Comparison of enzyme activity and sugar and starch contents between TQ and *sms2*. (**A**–**C**). Activities of three invertase isoforms in young panicles of WT and *sms2*. (**D**–**G**). Glucose, fructose, sucrose and starch contents in young panicles of WT and *sms2*. Data are mean ± S.D. ** *p* < 0.01; significant differences were based on 2-tailed *t*-test. ns indicates no significant difference. Red and blue dots indicate individual values of TQ and *sms2*, respectively.

To explore the physiological mechanism by which *SMS2* regulates grain size, we determined the changes in CIN, VIN, and CWIN enzyme activities and sugar and starch contents in TQ and *sms2* young panicles. CIN and VIN activities were significantly reduced in *sms2* compared to in TQ young panicles, but CWIN activity was not significantly different between the two groups (Figure 5A,B). In terms of sugar composition, *sms2* had considerably lower sucrose and fructose contents than TQ but no significant difference in glucose content (Figure 5D–F). The starch content of *sms2* was significantly lower than that of TQ (Figure 5G). These results suggest that *SMS2* may regulate grain size by reducing invertase activity and sucrose and fructose contents.

### 2.5. Analysis of SMS2 Gene Haplotype

**Figure 6:**
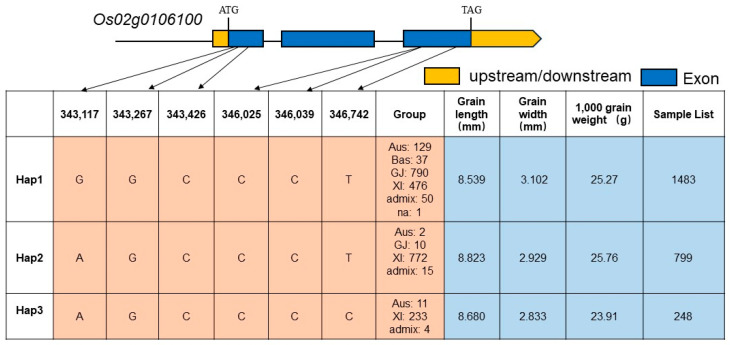
Haplotype analysis of *SMS2* gene (*Os02g0106100*). GJ—*Japonica* rice; XI—*Indica* rice.

In order to explore the variation characteristics of the *SMS2* gene haplotype and whether it plays a role in the differentiation of grain size between *indica* and *japonica*, we analyzed the *SMS2* haplotype using the RFGB website. We found six SNP mutations in the *Os02g0106100* gene exon region, totaling 13 haplotypes. We analyzed the haplotype variation characteristics of the top three samples in the Sample List. Among them, Hap2 had the longest grain length and the largest 1000-grain weight, compared with Hap1 and Hap3. It is worth noting that Hap2 is mainly found in *indica* rice (772 Sample List), but only 10 instances were found in *japonica* rice, showing the characteristics of differentiation between *indica* and *japonica*. The results of the statistical analysis showed that there were significant differences in grain length and width between Hap2 and Hap1 (Figure 6). Therefore, these results indicate that the *SMS2* gene may be responsible for the grain size differences between indica and japonica rice subspecies, and the Hap2 haplotype of *SMS2* is the dominant type, which can be used in the future cultivation of long-grain *japonica* rice varieties.

## 3. Discussion

Grain size is one of the critical agronomic traits that determine crop yield. Several key genes that regulate grain size and weight have been identified, but the molecular mechanisms that regulate grain size still remain unclear [51]. Therefore, it is necessary to identify more grain shape genes, enrich grain shape regulatory networks, and apply them to breeding practice. In this study, a small mutant, *sms2*, was identified from the HZ mutagenesis population of indica rice. The candidate gene was identified as *LOC_Os02g01590* using MutMap. Multiple alleles of this gene have previously been reported. The alleles of OsINV3 [46,48] and OsVIN2 [47] show a lower grain size and yield. In this study, in addition to significant reductions in grain length, width, and weight, the *SMS2* mutant also showed significant reductions in plant height and panicle number. Therefore, we speculate that *SMS2* is a strong allelic mutant of LOC_Os02g01590.

INV plays an important role in primary metabolism in rice. *OsVIN2* can regulate the grain size of rice by affecting sucrose metabolism [47]. *OsVIN2* also promotes sucrose transport to developing grains [52]. *OsCyt-inv1* regulates sucrose transport and controls root growth [53]. A previous study found that a decrease in *GhVIN1* expression in seed coat was the main reason for the decrease in female flower fertility in cotton [54]. Several studies have reported that INV can increase seed and fruit yields by facilitating the transport of sucrose from source to reservoir. A previous study found that increased CWIN activity in *Arabidopsis thaliana* accelerated flowering and increased seed yield by nearly 30% [55]. In cassava, *MeCWINV3* regulates the distribution of sugar from source to reservoir and influences the yield of storage roots by maintaining the sugar balance [56]. A previous study found that *SbVIN1* was significantly correlated with the grain traits of sorghum, such as 100-grain weight and grain width [57]. We also found that the VIN enzyme encoded by *SMS2* has a significant relationship with rice grain yield, as it can reduce the activity of the enzyme and the accumulation of sucrose and fructose in the grain (Figure 5), thus forming small grains.

Grain size is regulated by many signaling pathways, such as the transcription factor pathway, hormone processes, and the ubiquitin–protease pathway [2,58,59]. In order to further explore the regulatory mechanism of *SMS2* in grain size, RNA-seq was performed on the young panicles (5 cm) of HZ and *sms2*. The Gene Ontology (GO) terms of these DEGs were found to be significantly enriched in heat stress response, growth and development regulation, and cell proliferation regulation (Figure 4A). A KEGG analysis showed that many genes were enriched in endoplasmic reticulum protein synthesis, cysteine and methionine metabolism, propanoate metabolism, etc. (Figure 4B). These results suggest that *SMS2* regulates grain size via its involvement in multiple metabolic and developmental pathways, and further exploration is needed.

Evolutionary and functional analyses suggest that the grain shape genes *GW5/GSE5* [60], *GSE9* [61], *GS5* [12], *OsSPL13* [62], and *OsSPL12* [23] are involved in the regulation of grain shape differentiation between *indica* and *japonica* rice subspecies. A haplotype analysis showed that *SMS2* superior alleles were mainly selected and applied in *indica* rice, and they had good yield performance (Figure 6). The excellent haplotype Hap2 of *SMS2* may be a characteristic of *indica* and *japonica* differentiation, and it has not yet been used in *japonica* rice breeding. In the future, we can introduce the excellent allele of *SMS2* in *indica* rice into *japonica* rice via molecular-marker-assisted or gene-editing technology to create long-grain and high-quality *japonica* rice varieties.

In this study, we found that *SMS2* regulates rice grain size through a complex regulatory network, reduces sugar and starch accumulation in young panicles, affects various metabolic pathways, regulates grain size and yield, and has genetic effects on agronomic traits such as plant height and effective panicle number. The challenge ahead is to explore the upstream and downstream genes of *SMS2* regulators and identify the links between the various regulatory pathways. We need to accelerate the identification of the upstream and downstream factors of the *SMS2* gene and their interacting proteins, as this will help clarify the molecular mechanism by which *SMS2* regulates rice morphological development. In addition, it is worth noting that the genetic effects of the same gene on rice grain yield and quality in different genetic backgrounds are not consistent. One possible reason for this is the specific interactions between other major functional genes and *SMS2*. This makes it impossible to accurately assess the genetic effects of *SMS2* on rice yield and quality. Currently, genome-editing technology provides a way to generate mutants and specific mutant alleles in the same genetic context. These are ideal materials for in-depth analyses of the molecular regulation mechanism of grain shape, and they can help to elucidate the genetic effects of genes that regulate grain shape, such as *SMS2*. In addition, targeting and knocking out the *SMS2* gene using CRISPR technology can effectively create the excellent haplotype Hap2 in the japonica rice background. This opens up a new way to accelerate the breeding of high-quality and high-yield japonica rice germplasm resources.

## 4. Materials and Methods

### 4.1. Plant Material and Phenotypic Survey

The wild-type material was the *indica* rice Teqing (TQ), selected by the Guangdong Academy of Agricultural Sciences. *SMS2* is a small-grain mutant obtained via ethyl methane sulfonate (EMS) mutagenesis. We selected the small-grain mutant *sms2* from the M_3_ generation. The *sms2* mutant was crossed with the indica rice TQ to generate an F_2_ segregation population for gene mapping. All rice materials were grown in a field environment at the Sanya Southern Breeding Institute of Hainan University and the Institute of Genetics and Genetic Developmental Biology of the Chinese Academy of Sciences during 2022. At the maturity stage, 10 plants were selected to measure the effective panicle number and plant height, and the 10 main panicles were selected to measure yield traits.

### 4.2. Morphological Analysis of Rice Grain Size

Mature rice seeds were collected, and full rice seeds were selected for a phenotypic investigation. First, Scan Marker i560 (Microtek) was used to scan each material (seed number greater than 60), and then, the length and width of the seeds were measured using an SC-G seed analysis instrument. The 1000-grain weight was measured using an analytical balance (Mettler, AL104). All the above traits were measured ten times.

### 4.3. Genetic Analysis and Gene Mapping

The wild-type TQ was crossed with the small mutant SMS2 as the maternal parent to obtain 10 F_1_ seeds. In 2021, eight F1 plants were planted at the Institute of Genetics and Development of the Chinese Academy of Sciences in Lingshui, Hainan Province. To confirm true hybrids, the grain sizes of the F_1_ plants were observed. A total of 500 strains of the F_2_ population were planted in the experimental base of the Institute of Genetics and Development of the Chinese Academy of Sciences in Lingshui, Hainan Province, in 2022. The mutant and wild-type phenotypes of the F_2_ populations were counted, and the isolation rate was measured.

### 4.4. RNA Extraction, Library Construction, and Sequencing

At the heading stage, TQ and SMS with a young spikelet hull length of 5 cm-10 cm were sampled and collected 3 times, quickly placed in tin foil, frozen in liquid nitrogen, and then stored in a refrigerator at −80 °C until further use. For RNA-seq, the total RNA of the fresh young spikelet hulls was extracted using Trizol, according to the manufacturer’s protocol. Three biological replicates were used for each sample. A total of 2.0 μg of RNA per sample was used to construct cDNA libraries using an mRNA-seq V3 Library Prep Kit (Vazyme, Nanjing, China), according to the manufacturer’s instructions. A Bioanalyzer 2100 (Agilent, Palo Alto, CA, USA) was used to quantify and assess the quality of the RNA samples and cDNA libraries. Paired-end 2 × 150-base sequencing was performed on an Illumina HiSeq X sequencing platform.

### 4.5. Analysis of Differentially Expressed Genes

A cluster analysis can be used to study the expression patterns of differential genes under different experimental conditions. Similar genes may have the same function and participate in the same metabolic process or cellular pathway. Genes with the same or similar expression patterns can be clustered to predict the function of unknown genes or new functions of known genes. After read count data were standardized using DESeq, a gene differential expression analysis was performed (*p* < 0.05); the overall distribution of the differential genes was inferred using volcano maps, and the expression level was determined via the FPKM value of the differential genes. Then, a cluster analysis diagram was constructed.

### 4.6. GO and KEGG Enrichment Analyses

In order to clarify the main biological functions and biochemical metabolic pathways of *SMS2* in the regulation of seed size, the clusterprofile of the R software package R-4.3.2 was used to perform GO functional enrichment and KEGG pathway analyses of genes with differential expression in the TQ and *sms2* young panicles. The databases on (http://geneontology.org/, accessed on 2 September 2023) were used for the GO enrichment analysis, and the databases on (https://www.genome.jp/kegg/, accessed on 10 September 2023) were used for the KEGG enrichment analysis. Functional enrichment maps of the GO/KEGG analyses of differentially expressed genes were generated using R software.

### 4.7. Analysis of Invertase Activity and Sugar and Starch Contents

At the heading stage, the HZ and *sms2* young panicles (5–10 cm) were taken to determine the sugar and starch contents and enzyme activity. The measurements were repeated three times for all three sugars and three enzymes. The detection of the starch content was mainly carried out by referring to the method for determining rice quality according to standard NY/T 83-2017 of the Ministry of Agriculture. For descriptions of the specific methods, please refer to [48].

### 4.8. Haplotype Analysis of SMS2 and Its Distribution in Indica and Japonica Rice

Through the haploid-type analysis module in the RFGB database (https://www.rmbreeding.cn/Index/manual, accessed on 1 October 2023), we analyzed the *SMS2* mutation information in the exon region, revealing 13 different haplotypes. We analyzed the phenotypic data of grain size and distribution in the germplasm resources of the three most common haplotypes.

In order to study the distribution of the *SMS2* gene in indica and japonica rice, we focused on the number and percentage of the three most common haplotypes in *SMS2* in indica and japonica rice. Grain length, grain width, and 1000-grain weight were analyzed to estimate the superiority of the *SMS2* haplotype and its application in breeding.

### 4.9. Statistical Analysis

All data were collected from at least ten independent biological replicates, and they are presented as means ± SD. A statistical analysis was performed using SPSS13.0 software. Statistical significance was determined using Student’s *t*-test for a comparison of the two groups. Graphs were created using Adobe Photoshop CC 2020 and GraphPad Prism 6.0.

## 5. Conclusions

In this study, a small-grain mutant, SMS2, was identified from an EMS mutagenesis population of the indica rice TQ. Compared with TQ, the grain length, grain width, and 1000-grain weight of the SMS2 mutant were significantly reduced. MutMap and sequencing analysis showed that the candidate gene was the *LOC_Os02g01590* gene. Due to the 2 bp insertion in the second exon of the *LOC_Os02g01590* gene in the *sms2* mutant, the resulting frameshift mutations formed a truncated SMS2 protein. We found that *SMS2* encoded vacuolar acid invertase, a novel allele of *OsINV3*, which regulates grain size. GO and KEGG enrichment analyses showed that *SMS2* was involved in endoplasmic reticulum protein synthesis, cysteine and methionine metabolism, and propionic acid metabolism, thereby regulating grain size. The activity of vacuolar acid invertase and the contents of sugar and starch indicated that *SMS2* might regulate grain size by regulating the metabolic accumulation of sucrose and starch in the grain. A haplotype analysis showed that *SMS2* might be a differentiation gene in indica and japonica, and it has great application value in japonica rice breeding. This study further enriches the mutant materials related to grain shape and provides gene resources for further understanding of the genetic regulatory network of grain shape in rice.

## Data Availability

All data and conclusions are included in this paper.
